# High-concentration zeta potential measurements using light-scattering techniques

**DOI:** 10.1098/rsta.2010.0175

**Published:** 2010-09-28

**Authors:** Michael Kaszuba, Jason Corbett, Fraser Mcneil Watson, Andrew Jones

**Affiliations:** Malvern Instruments Ltd, Grovewood Road, Enigma Business Park, Malvern, Worcestershire WR14 1XZ, UK

**Keywords:** zeta potential, electrophoretic mobility, electrophoretic light scattering, high concentration, high turbidity, concentration effects

## Abstract

Zeta potential is the key parameter that controls electrostatic interactions in particle dispersions. Laser Doppler electrophoresis is an accepted method for the measurement of particle electrophoretic mobility and hence zeta potential of dispersions of colloidal size materials. Traditionally, samples measured by this technique have to be optically transparent. Therefore, depending upon the size and optical properties of the particles, many samples will be too concentrated and will require dilution. The ability to measure samples at or close to their neat concentration would be desirable as it would minimize any changes in the zeta potential of the sample owing to dilution. However, the ability to measure turbid samples using light-scattering techniques presents a number of challenges. This paper discusses electrophoretic mobility measurements made on turbid samples at high concentration using a novel cell with reduced path length. Results are presented on two different sample types, titanium dioxide and a polyurethane dispersion, as a function of sample concentration. For both of the sample types studied, the electrophoretic mobility results show a gradual decrease as the sample concentration increases and the possible reasons for these observations are discussed. Further, a comparison of the data against theoretical models is presented and discussed. Conclusions and recommendations are made from the zeta potential values obtained at high concentrations.

## 1. Introduction

**Figure 1.**
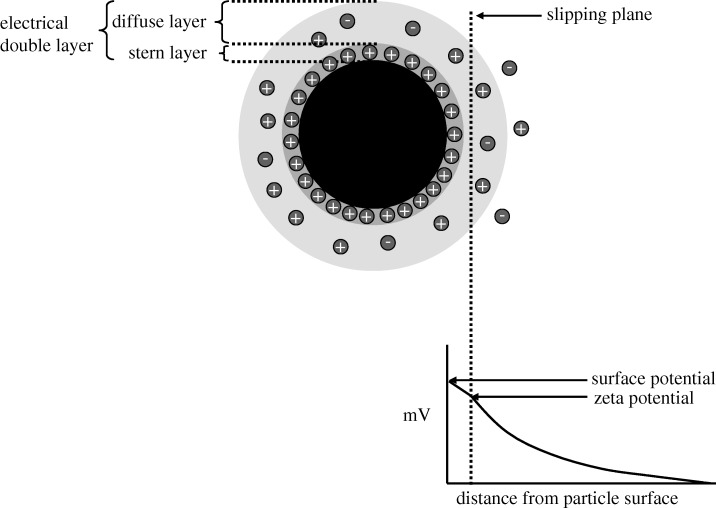
Schematic showing the electrical double layer that surrounds a particle in an aqueous medium and the position of the slipping plane. The zeta potential is the electrical potential at the slipping plane.

Zeta potential is the key parameter that controls electrostatic interactions in particle dispersions and, as such, it is important in understanding the stability of colloidal dispersions. It can be used to optimize the formulations of suspensions and emulsions and aid in predicting long-term stability (Shaw 1992; Hunter 1993; Everett 1994). Most colloidal dispersions in aqueous media carry an electric charge and the development of this charge at the particle surface affects the distribution of ions in the surrounding interfacial region. An increase in the concentration of counter ions close to the surface results in the formation of an electrical double layer. The liquid layer surrounding the particle (the electrical double layer) exists as two parts—an inner region (Stern layer) where the ions are strongly bound and an outer (diffuse) region where they are less firmly associated. Within the diffuse layer there is a notional boundary inside which the ions and particles form a stable entity. When a particle moves (e.g. owing to electrophoresis), ions within the boundary move with it. Those ions beyond the boundary stay with the bulk dispersant. The potential at this boundary (surface of hydrodynamic shear or slipping plane) is the zeta potential (figure 1; Hunter 1988; Lyklema 2000; Delgado *et al.* 2005).

The zeta potential of colloidal dispersions is routinely measured using the technique of micro-electrophoresis (Washington 1992; Johnson & Gabriel 1994; Delgado *et al.* 2005). In this technique, a voltage is applied across a pair of electrodes at either end of a cell containing the particle dispersion. Charged particles are attracted to the oppositely charged electrode and their velocity is measured and expressed in unit field strength as their electrophoretic mobility. Light scattering is one of the most commonly used techniques for determining the electrophoretic mobility of particles. Laser Doppler electrophoresis measures small frequency shifts in the scattered light that arise owing to the movement of particles in an applied electric field. The frequency shift *Δf* is equal to

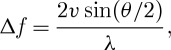

where *ν* is the particle velocity, *λ* is the laser wavelength and *θ* is the scattering angle. The measured electrophoretic mobility (*U*_E_) is converted into zeta potential (*ζ*) through Henry’s equation,

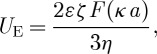

where *ε* is the dielectric constant of the dispersant, *F*(*κa*) is the Henry function and *η* is the viscosity (Hunter 1988; Delgado *et al.* 2005). This equation strictly only applies for isolated particles of zeta potential less than around 25 mV. A simpler form in which *F*(*κa*)=1.5 is known as the Smoluchowski equation (Smoluchowski 1921), and applies where *κa* is large (approx. 100) and the double layer is thin in comparison with the particle radius. This limit is relevant to some but not all of the sample measurements presented in this paper. Hence the data are given in the form of mobility, and the more complex formulations needed to deal with the derivation of zeta potential, where the particle is not isolated and the zeta potential not small, are addressed in a later section.

**Figure 2.**
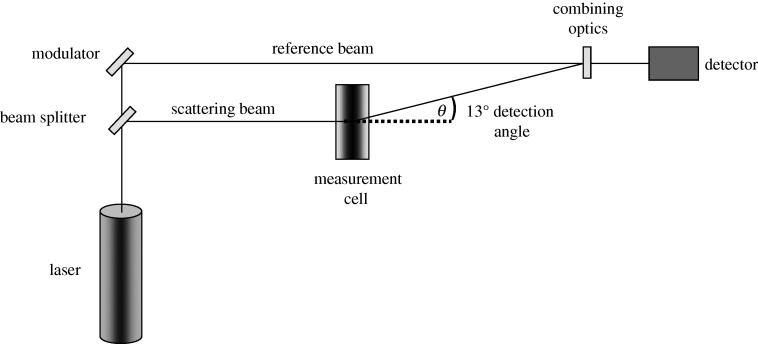
Schematic of typical optical configuration for a laser Doppler electrophoresis instrument.

In a typical optical configuration used for electrophoretic mobility (and hence zeta potential) measurements, the laser beam has to penetrate the sample for scattered light at a forward angle of 13^°^ to be detected (figure 2). This optical configuration imposes restrictions on the maximum turbidity of the sample which can be measured. If the concentration of the sample is too high, the laser beam will become attenuated by the particles reducing the scattered light that is being detected. At very high concentrations, no scattered light is detected. The upper limit of concentration for zeta potential measurements will depend upon a number of factors such as the particle size, polydispersity and optical properties of the particles.

One way to measure the electrophoretic mobility of samples at higher concentration is to reduce the path length of the cell, which will increase the transmission of the laser through the sample. Even though such a cell will allow electrophoretic mobility measurements to be made on concentrated samples, the conversion to zeta potential, and subsequent interpretation of what the results obtained mean, is not straightforward.

This paper summarizes measurements made on different types of samples at high concentration and turbidity and discusses the concentration dependence on the zeta potential values obtained.

## 2. Experimental

All measurements reported in this paper were made at a temperature of 25^°^C on a Zetasizer Nano ZS (Malvern Instruments Ltd, Malvern, UK) fitted with a high-concentration zeta potential cell (ZEN1010).

Two samples were measured in this study. (i) A titanium dioxide (anatase) sample was prepared in 10 mM NaCl at a range of volume fractions (0.236×10^−5^ to 7.092×10^−4^). Dilution in an indifferent electrolyte, such as NaCl, should ensure that any changes in the zeta potential values obtained were not due to conductivity differences. (ii) A polyurethane dispersion at a volume fraction of 0.4 was kindly provided by Baxenden, a Chemtura company. Various concentrations were prepared in 5 per cent v/v triethylamine to maintain ionic strength and ensured that any differences in the measured electrophoretic mobilities were not due to changes in conductivity.

For all measurements, a field of 40 V was applied across an electrode spacing of 16 mm. Five repeat measurements on each sample were made to check the repeatability of the results obtained. All measured electrophoretic mobilities were converted into zeta potential using Smoluchowski’s formula (Smoluchowski 1921; Delgado *et al.* 2005). Sample viscosities were determined at 25^°^C using an SV-10 vibroviscometer (A&D Company Ltd, Tokyo, Japan).

Size characterization of the samples was made by dynamic light-scattering (DLS) measurements using the Zetasizer Nano ZS, which uses a 4 mW He–Ne laser operating at a wavelength of 633 nm and a detection angle of 173^°^. The intensity-averaged particle diameters and the polydispersity index (PDI) values (an estimate of the distribution width) were calculated from the cumulants analysis as defined in ISO13321 (International Organization for Standardization 1996). The intensity size distributions were obtained from analysis of the correlation functions using the general purpose algorithm in the instrument software. This algorithm is based upon a non-negative least squares fit (Lawson & Hanson 1995; Twomey 1997).

## 3. Results

### (a) Titanium dioxide

**Figure 3.**
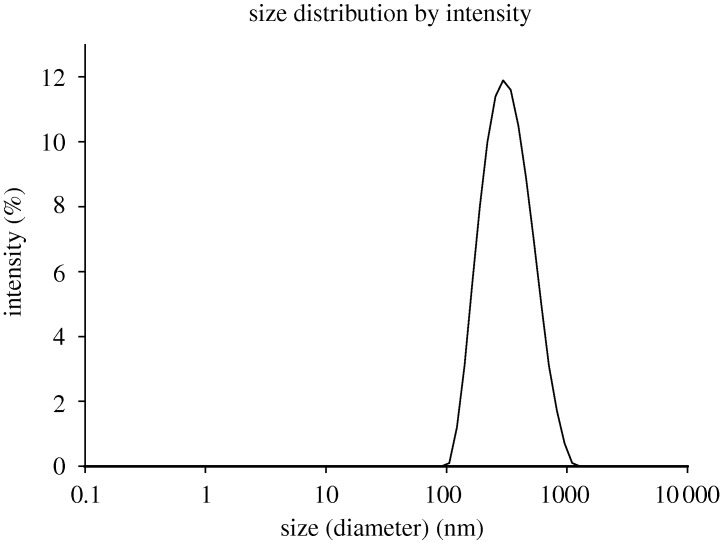
Intensity particle size distribution of a TiO_2_ sample (volume fraction of 2.36×10^−4^) dispersed in 10 mM NaCl measured on a Zetasizer Nano ZS.

Figure 3 is the intensity particle size distribution obtained from the DLS measurements made on a Zetasizer Nano ZS. The intensity-weighted mean diameter was 295 nm with a polydispersity index of 0.18.

**Figure 4.**
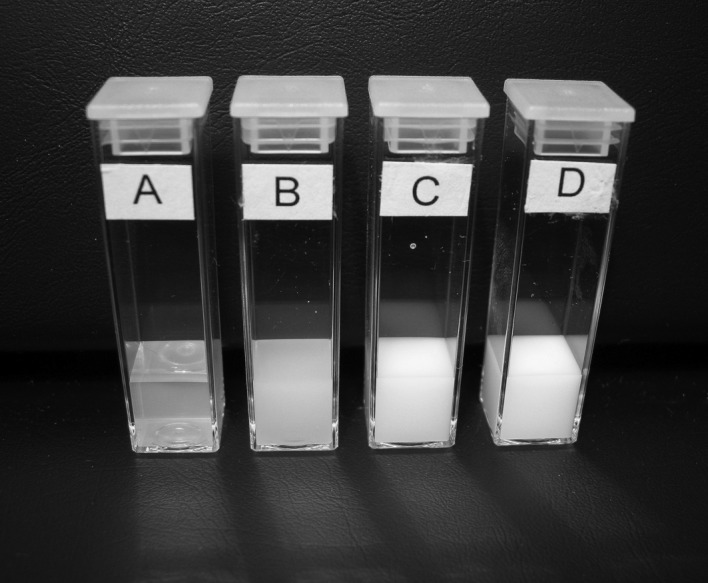
Photograph of cuvettes containing various volume fractions (*A*=2.36× 10^−5^, *B*=2.36×10^−4^, *C*=4.728×10^−4^ and *D*=7.092×10^−4^) of the TiO_2_ dispersed in 10 mM NaCl.

Figure 4 shows cuvettes containing various volume fractions of titanium dioxide dispersed in 10 mM NaCl measured in the high-concentration zeta potential cell in this study. Even though the higher volume fractions show very high turbidity, their electrophoretic mobilities have still been successfully measured in the ZEN1010 high-concentration cell.

**Figure 5.**
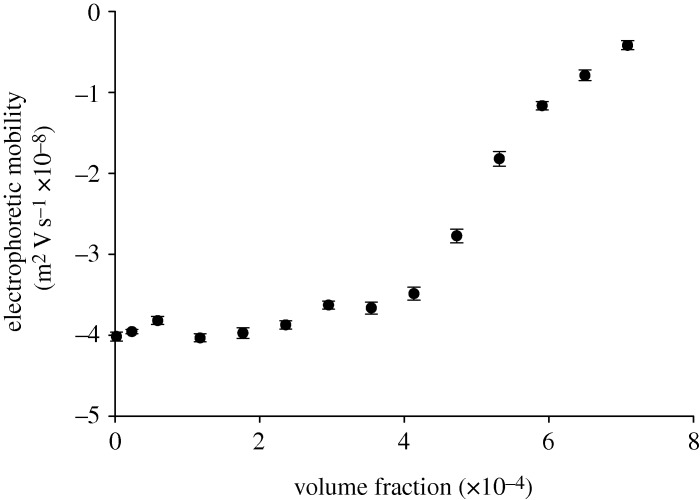
Electrophoretic mobilities (m^2^V s^−1^×10^−8^) as a function of various volume fractions of TiO_2_ samples dispersed in 10 mM NaCl.

Table 1 summarizes the electrophoretic mobility values obtained for various volume fractions of the TiO_2_ sample prepared in 10 mM NaCl. The table also includes the measured conductivity values for these samples and illustrates that the presence of 10 mM NaCl maintains a consistent ionic strength across the concentration range. The standard deviation values are obtained from the five repeat measurements performed on each sample. The electrophoretic mobility values are plotted as a function of volume fraction in figure 5.

**Table 1.** Electrophoretic mobility results obtained for various volume fractions of TiO_2_ samples dispersed in 10 mM NaCl.

	electrophoretic mobility	standard deviation	conductivity
volume fraction	(m^2^V s^−1^×10^−8^)	(mV)	(mS cm^−1^)
0.236×10^−5^	−4.018	0.055	0.97
0.236×10^−4^	−3.957	0.025	0.99
0.591×10^−4^	−3.819	0.049	1.02
1.182×10^−4^	−4.033	0.051	0.99
1.773×10^−4^	−3.972	0.067	0.97
2.364×10^−4^	−3.873	0.052	1.01
2.955×10^−4^	−3.628	0.051	0.98
3.546×10^−4^	−3.665	0.074	0.99
4.137×10^−4^	−3.489	0.082	1.02
4.728×10^−4^	−2.775	0.085	1.01
5.319×10^−4^	−1.820	0.090	1.01
5.910×10^−4^	−1.166	0.049	1.01
6.501×10^−4^	−0.790	0.065	1.01
7.092×10^−4^	−0.420	0.055	0.98

### (b) Polyurethane dispersions

**Figure 6.**
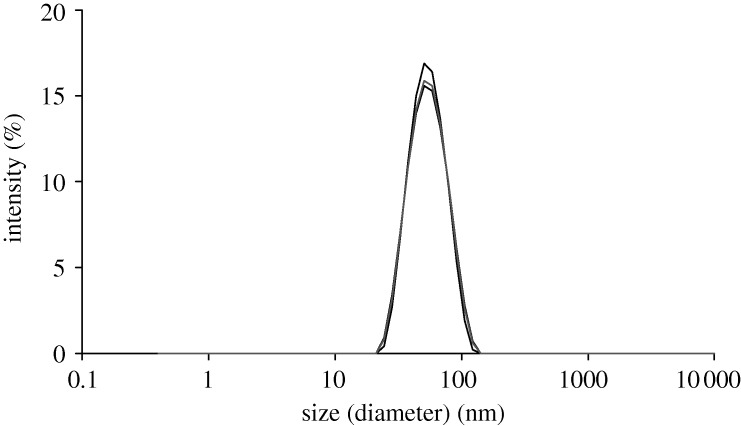
Intensity particle size distributions obtained for the 0.01 volume fraction polyurethane dispersion sample prepared in 5% v/v triethylamine.

The intensity particle size distributions obtained from the three repeat measurements are over plotted in figure 6 and highlight the repeatability of the results obtained. The samples had a mean intensity-weighted diameter of 50.5 nm with a PDI value of 0.101.

**Figure 7.**
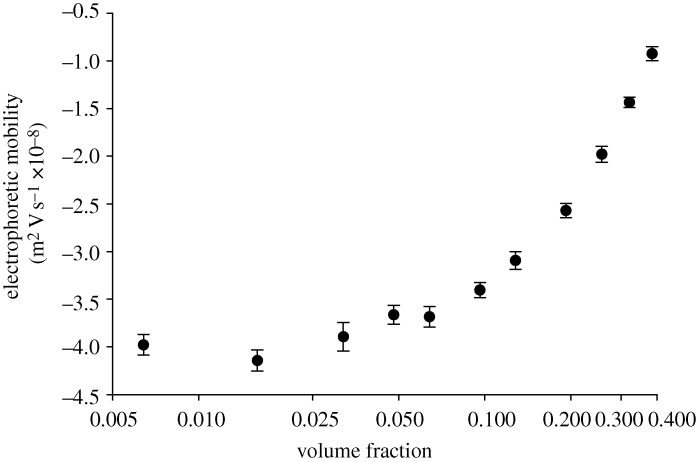
The electrophoretic mobilities (m^2^V s^−1^×10^−8^) measured for the polyurethane dispersions as a function of the volume fraction of the sample.

Figure 7 is a plot of the electrophoretic mobilities (m^2^ V s^−1^×10^−8^) measured as a function of volume fraction for the polyurethane dispersions dispersed in 5 per cent v/v triethylamine (apart from the neat sample with a volume fraction of 0.4).

## 4. Discussion

### (a) Titanium dioxide

The optical configuration used in a typical laser Doppler instrument such as the Zetasizer Nano means that the laser beam has to penetrate the sample for scattered light to be detected at a forward angle. Therefore, in general, samples for electrophoretic mobility measurements have to be optically clear. As discussed above, the maximum concentration at which the electrophoretic mobility of a sample can be measured will depend upon a number of factors such as particle size, polydispersity of the particles and optical properties. The optical properties (refractive index=2.4) and particle size distribution (figure 3) of titanium dioxide result in very high scattering, which produces highly turbid concentrations (figure 4).

The high-concentration zeta potential cell, with its reduced path length, allows electrophoretic mobility measurements to be made on samples which are turbid and at high concentration. However, the influence of concentration on the mobility results obtained needs to be studied and understood. In the results summarized in table 1 and figure 5, the measured mobility values are consistent over a wide range of sample concentrations. The results obtained over the 2.34×10^−5^ to 4.137×10^−4^ volume fraction range have a mean value of −48.9 mV with a standard deviation of 2.5 mV. However, as the concentration increases above a volume fraction of 4.137×10^−4^, the mobility values become less negative, i.e. the magnitude of mobility decreases.

There are various possibilities for the concentration dependence in the mobilities obtained. Dilution may have led to a change in sample equilibrium. This is unlikely as the samples were prepared in a non-specific electrolyte of sufficient concentration (10 mM NaCl) to maintain consistent conductivity for each dilution (table 1). The effect of sample viscosity can also be discounted as, even at the highest concentration measured (a volume fraction of 7.09×10^−4^), the viscosity was determined to be that of water (0.89 mPa.s at 25^°^C).

The most probable explanation is that the increase in sample concentration leads to higher turbidity, resulting in obscuration of light transmission. Therefore, the scattered light being detected originates from particles close to the wall, rather than the centre, of the cell where they have reduced mobility owing to the electric field being lower than assumed.

### (b) Polyurethane dispersions

The polyurethane dispersion used in this study is ideally suited for looking at the influence of concentration on the electrophoretic mobility and hence zeta potential. Measurements can be made even at the neat concentration of 0.4 volume fraction with the high-concentration zeta potential cell owing to the particle size of around 50 nm and the low polydispersity index values.

Figure 7 shows the electrophoretic mobility values obtained as a function of sample concentration. The values obtained at low concentrations are consistent (around −4.0 m^2^ V s^−1^×10^−8^). However, at concentrations above a volume fraction of 0.1, there is a gradual decrease in the electrophoretic mobilities measured. This is not unexpected as the viscosity of the sample increases significantly with concentration.

**Figure 8.**
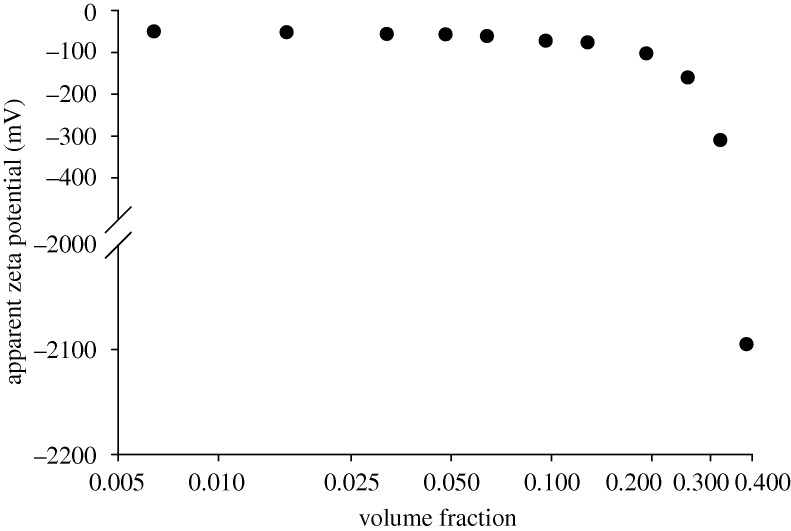
Apparent zeta potential values (mV) calculated from the Smoluchowski relationship as a function of the volume fraction of the sample using the viscosity values measured on the SV-10 vibroviscometer.

Henry’s equation relates the electrophoretic mobility to zeta potential through Henry’s function, the dielectric constant of the dispersant and the sample viscosity. In this study, an *F*(*κa*) of 1.5 (i.e. Smoluchowski’s approximation) and the dielectric constant of water were used. The viscosities of the samples measured on the SV-10 vibroviscometer were used to calculate the zeta potential values. The zeta potential results obtained from these calculations are plotted as a function of sample concentration in figure 8.

The zeta potential values at the higher sample concentrations are not realistic, indicating that the viscosity values obtained from a simple viscometer such as the SV-10 vibroviscometer cannot be used. The macroscopic viscosity measured by the viscometer is probably not representative of the local viscosity which the particles experience as they undergo electrophoresis. These polyurethane dispersions are complex samples probably exhibiting non-Newtonian behaviour and, therefore, it is uncertain which viscosity value is the most appropriate to use for the conversion of the electrophoretic mobility data into zeta potential values.

**Figure 9.**
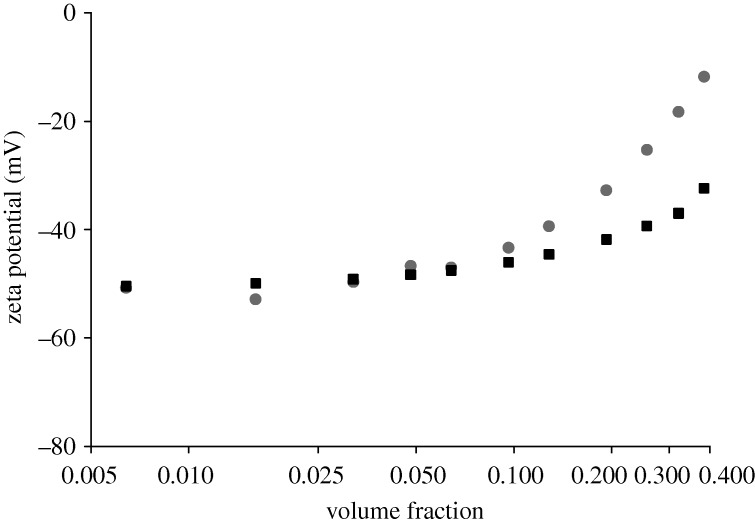
Zeta potential values (mV) calculated as a function of the volume fraction of the sample using the viscosity values of water (filled circles) and the Anderson relationship (filled squares).

If the viscosity is assumed to be that of water for all volume fractions of the sample, the zeta potential values obtained are more realistic (figure 9). However, there is still a concentration dependence in the values above a volume fraction of 0.1. It is difficult to understand the effects which are causing this behaviour. Apart from the viscosity problems and non-Newtonian behaviour of the sample, the average particle spacing becomes very small at high concentrations. At a volume fraction of 0.4, for example, the average particle spacing is of the order of 56 nm. The thickness of the electrical double layer (the Debye length) can be calculated from the ionic strength of the dispersant (Lyklema 2000; Delgado *et al.* 2005). Theoretically, in 5 per cent triethylamine, it will be of the order of a few nanometres and would not be expected to greatly influence the electrophoretic mobility of the particles. However, with such small average particle spacing at high concentrations, overlapping electrical double layers may have an influence.

### (c) Comparison of high-concentration experimental results with theory

There are relatively few references which discuss concentration dependence on electrophoretic mobility and even fewer exist that compare the predictions of these models with experimental evidence. Hunter (1988) dedicates a short section to the effects of particle concentration. The two primary models are those of Anderson (1981), which relies on a statistical description of particle–particle interactions at the microscopic level, and Ohshima (1997), who extends the work of Levine & Neale (1974), providing a model based on a Kuwabara (1959) cell model.

### (d) Anderson

The Anderson relationship between the electrophoretic mobility *μ* at volume fraction *φ* and the mobility at infinite dilution, *μ*_0,_ is given by

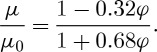

The electrophoretic mobility values obtained for the various sample concentrations can therefore be ‘corrected’ using this relationship (figure 9). This relationship shows a much stronger correlation with experiment than the viscosity correction seen in figure 8. However, significant deviations of the model from experiment still occur at volume fractions above 0.1.

### (e) Ohshima–Levine–Neale

Ohshima extends the work of Levine and Neale to a more general case to include the work of Kozak & Davis (1989*a*,*b*). Their models are based on a Kuwabara cell model, whereby each particle is considered to be surrounded by a virtual cell whose volume ratio is equal to that of the volume fraction of the entire sample and whose fluid vorticity is zero at the outer surface of the cell.

**Figure 10.**
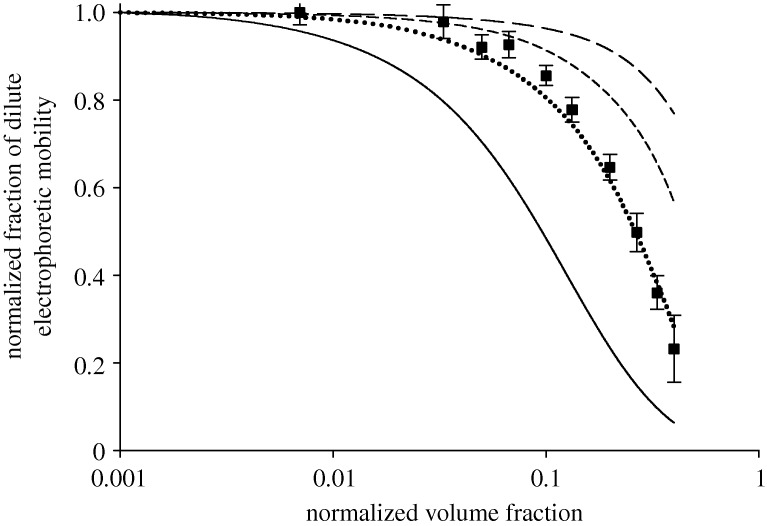
Reported mobility as a fraction of the dilute limit for both the polyurethane dispersion data and the Ohshima–Levine–Neale model (long-dashed line, Ohshima *κa*=10; short-dashed line, Ohshima *κa*=5; dotted line, Ohshima *κa*=2.5; solid line Ohshima *κa*=1; filled square, polyurethane dispersion data).

Despite the apparent fit to the *κa*=2.5 curve above a normalized volume fraction of 0.1, the *κa* value for this sample is computed as approximately 5. From figure 10, significant deviation of the data from the *κa*=5 curve occurs at about a volume fraction of 0.07 in much the same region as the Anderson model. It is far beyond the scope of this paper to discuss the comparison of the two models with the experimental data in great detail. For now, the curiosity that they both fail at the same concentration despite the obvious differences in approach is noted here. Effects not modelled in either case include the viscoelectric effect and changes in the electrochemical potential of the ions in the system owing to the higher volume fraction, although it is acknowledged that the similar point of failure may simply be coincidental. A more rigorous study will be reported at a later date.

It is important to note, however, that, despite the discrepancies between data and model above volume fractions of 0.1, the small particle size and low refractive index of this sample mean that the measured change in mobility can, in this case, be taken as real and not an artefact of the measurement technique.

## 5. Conclusions

The results presented in this paper confirm that high-concentration zeta potential measurements of samples up to volume fractions of 0.4 can be made using light-scattering techniques when a suitable optical configuration, such as a cell of reduced path length, is used. The maximum measurable concentration of a sample will depend on the mean particle size, the polydispersity of particles and the optical properties.

The results obtained often show a concentration dependence and such trends need to be carefully understood and interpreted. For both of the sample types discussed in this paper, the electrophoretic mobility results show a gradual decrease as the sample concentration increases. However, this trending in the electrophoretic mobility data occurs for different reasons for each sample. For the titanium dioxide measurements, the reduction is an optical effect owing to the increasing turbidity of the sample, which results in an increase in the obscuration of transmitted light. For the polyurethane dispersions, the trend is not due to an optical effect as the intensity of scattered light being detected is still very high even at volume fractions of 0.4.

The conversion of the measured electrophoretic mobility values into zeta potential using Henry’s equation requires the viscosity of the sample to be entered. In a complex sample such as the polyurethane dispersion used in this study, it is difficult to know what the most appropriate viscosity value to use is. The results summarized in figures 8 and 9 show that assuming the viscosity of water results in zeta potential values which are more realistic than the use of bulk viscosities measured on a simple viscometer.

From the results obtained in this study, it can be concluded that zeta potential values obtained at high concentrations should be used in a relative, not absolute, sense. If the true zeta potential value of a sample is required, measurements should be performed at a range of concentrations to identify a region where the zeta potential results obtained are independent of concentration. For these experiments, the dilution protocol is pivotal to ensuring that sample conditions are kept constant.

Two models of mobility were tested against the experimental data collected and shown to provide a good estimate of the change in mobility owing to high concentration below normalized volume fractions of about 0.1.
